# Different levels of overnutrition and weight gain during pregnancy have differential effects on fetal growth and organ development

**DOI:** 10.1186/1477-7827-8-75

**Published:** 2010-06-24

**Authors:** Lindsey A George, Adam B Uthlaut, Nathan M Long, Liren Zhang, Yan Ma, Derek T Smith, Peter W Nathanielsz, Stephen P Ford

**Affiliations:** 1 The Center for the Study of Fetal Programming, University of Wyoming, Laramie, Wyoming, USA; 2 Dept. of Animal Science, 1000 E. University Ave. Dept. 3684, University of Wyoming, Laramie, Wyoming, USA; 3 Division of Kinesiology and Health, 1000 E. University Ave. Dept. 3196, University of Wyoming, Laramie, Wyoming, USA; 4 Center for Pregnancy and Newborn Research, Department of Obstetrics and Gynecology, University of Texas Health Sciences Center, San Antonio, Texas, USA

## Abstract

**Background:**

Nearly 50% of U.S. women of child-bearing age are overweight or obese, conditions linked to offspring obesity and diabetes.

**Methods:**

Utilizing the sheep, females were fed a highly palatable diet at two levels of overfeeding designed to induce different levels of maternal body weight increase and adiposity at conception, and from conception to midgestation. Fetal growth and organ development were then evaluated at midgestation in response to these two different levels of overfeeding. Ewes were fed to achieve: 1) normal weight gain (control, C), 2) overweight (125% of National Research Council [NRC] recommendations, OW125) or 3) obesity (150% of NRC recommendations, OB150) beginning 10 wks prior to breeding and through midgestation. Body fat % and insulin sensitivity were assessed at three points during the study: 1) diet initiation, 2) conception and 3) mid-gestation. Ewes were necropsied and fetuses recovered at mid-gestation (day 78).

**Results:**

OB150 ewes had a higher % body fat than OW125 ewes prior to breeding (P = 0.03), but not at mid-gestation (P = 0.37). Insulin sensitivity decreased from diet initiation to mid-gestation (P = 0.04), and acute insulin response to glucose tended to be greater in OB150 ewes than C ewes (P = 0.09) and was greater than in OW125 ewes (P = 0.02). Fetal crown-rump length, thoracic and abdominal girths, and fetal perirenal fat were increased in the OW125 and OB150 versus C ewes at mid-gestation. However, only fetal heart, pancreas, and liver weights, as well as lipid content of fetal liver, were increased (P < 0.05) in OB150 ewes versus both C and OW125 ewes at midgestation.

**Conclusions:**

These data demonstrate that different levels of overfeeding, resulting in differing levels of maternal weight gain and adiposity prior to and during pregnancy, lead to differential effects on fetal overgrowth and organ development.

## Background

Approximately two-thirds of U.S. adults are overweight or obese [1]. Among women ages 20 to 44, approximately 25% are overweight and an additional 23% are obese [2]. With these rates of overweight/obesity and over four million births in the U.S. annually, approximately two million births are likely to occur from overweight or obese mothers each year. Maternal obesity has been linked to an increased rate of obese children and adolescents. When female offspring become overweight or obese, a self-perpetuating cycle of obesity and its related health problems is established [3-5]. In 2003-2004, rates of overweight children ages 2-5 yrs were 14% and at ages 6-11 yrs were 19%, increased markedly from rates of approximately 5% in similar age children reported in the 1970s [1].

Insulin resistance during pregnancy is a normal maternal adaptation which is thought to help direct nutrients, particularly glucose, to the feto-placental compartment. As pregnancy progresses, it is observed that insulin response to elevated blood glucose increases, while peripheral insulin sensitivity (the ability of insulin to accelerate glucose clearance from the blood into tissues) decreases [6,7]. Severe insulin resistance may result in hyperinsulinemia, hyperglycemia and eventual gestational diabetes, conferring risk to both mother and fetus [7-9].

Maternal obesity in pregnancy has been linked to increased fat deposition in fetal sheep [10]. In humans, neonates of obese mothers demonstrated increased adiposity, higher indices of insulin resistance (homeostasis model assessment) and a significant correlation between neonatal insulin resistance and maternal pre-pregnancy body mass index (BMI) [11]. Greater adiposity and altered glucose and insulin dynamics in fetal and neonatal life are mechanisms which may predispose offspring of obese mothers to obesity and metabolic disease later in life.

Moreover, pre-pregnancy BMI is associated with greater neonatal adiposity independent of birth weight or maternal weight gain during pregnancy in women [12]. Childhood obesity heightens childhood risk of metabolic syndrome, indicating that prevention of early onset obesity may substantially reduce the prevalence of metabolic syndrome in youth and their future risk of life-threatening conditions such as diabetes and cardiovascular disease [4].

The aim of this study was to examine the effects of two levels of maternal overfeeding initiated prior to conception and continuing through mid-pregnancy on maternal weight gain, % body fat, and glucose and insulin dynamics, in association with changes in fetal growth and organ development in the ewe.

## Methods

### Animals and dietary treatments

All methods were approved by the University of Wyoming Animal Care and Use Committee. Twenty nulliparous Western white-faced ewes (Rambouillet/Columbia breeding) were randomly divided into three dietary groups and fed a highly palatable diet at one of 3 levels: 1) fed to maintain body weight (allowing 10-15% increase in BW during early gestation; control, C; n = 7), 2) fed a global nutrient excess of 125% of National Research Council (NRC) recommendations [13] to become overweight (OW125, n = 8) or 3) fed a global nutrient excess of 150% of NRC recommendations to become obese (OB150, n = 5). Ewes were adapted from their previous diet of mixed legume-grass hay to the experimental diet (Table 1) at 100% of NRC recommendations over a one week period. Experimental diets given at appropriate treatment levels (C, OW125 and OW150) were then applied beginning in September for 10 wks prior to breeding and continued throughout the first half of gestation (February/March). Feed was provided to ewes once daily at approximately 1600 hr. Ewes were grouped into six adjacent pens in an open fronted pole barn. Each treatment group (C, OW125 and OB150) was divided into two pens per dietary treatment to allow replication. Feed amounts which were calculated based on body weight (BW) according to NRC guidelines were adjusted weekly to account for increases in BW. An intact ram (white-faced, Rambouillet/Columbia breeding) fitted with a marking harness was continuously maintained in each of the six pens for approximately six weeks beginning in late November, and the first day each ewe marked was considered day 0 of gestation.

**Table 1:** Nutrient analysis of experimental diet

	*Mean *	*SE *
**% Dry matter**	88.1	1.01
**% Crude protein**	9.2	0.20
**% Acid detergent fiber**	14.9	1.35
**% Neutral detergent fiber**	25.2	2.03
**% Total digestible nutrients**	73.7	0.80

Results are reported as a percent of dry matter.

### Ewe anthropometrics and blood collection

All ewes were weighed weekly, and body condition score (BCS) was obtained bi-weekly to detect changes in subcutaneous fat deposition. BCS was assessed independently by two trained evaluators by palpation of the spine, spinous processes, ribs and tail-head on a 1 (emaciated) to 9 (obese) scale as previously established for sheep [14]. An average score was then calculated from the two evaluators. Blood was collected bi-weekly between 0900 and 1100 h via jugular venipuncture into two blood collections tubes containing either no anti-coagulant or sodium heparin (143 U.S.P units per 10 mL whole blood, BD Vacutainer, Franklin Lakes, NJ). Heparinized tubes were immediately centrifuged at 1000 × g for 15 min and plasma frozen at -20°C until time of assay for glucose and insulin. Tubes without anti-coagulant were allowed to sit at room temperature for 1 hour, and then refrigerated at 4°C overnight. Serum was then collected after centrifugation (1000 × g for 15 min) the following morning, and stored frozen at -20°C until used for leptin assay.

### Dual Energy X-ray Absorptiometry (DEXA)

To accurately determine total % body fat, Dual Energy X-ray Absorptiometry (DEXA, GE Lunar Prodigy™ 8743; Madison, WI) was utilized as previously used in our laboratory and previously described and validated for sheep [15-17]. DEXA scanning was performed in a subset of 12 ewes (4 from each dietary group) at three different sample points: 1) immediately prior to diet initiation, 2) immediately prior to breeding and 3) at mid-gestation. Crown to rump length (CRL) of each ewe was measured and used in place of height to calculate sheep body mass index (BMI = (BW, kg)/(CRL, m)^2^). Ewes were deprived of food and water for approximately 24 h to prevent emesis and subsequent aspiration of gastric material while under sedation, and were sedated with Ketamine (22.2 mg/kg body weight) immediately prior to performing DEXA scans. The whole body scan mode was used for all animals and scan times were ~3 min depending on the length of the animal. A single, blinded, and experienced investigator performed all DEXA scans and quantified % body fat. DEXA was calibrated and quality assurance tests performed daily prior to measurement and according to the manufacture specifications and programmed acceptable limits.

### Intravenous glucose tolerance tests

An insulin-modified frequently sampled intravenous glucose tolerance test (FSIGT) was applied to the same subset of 12 ewes utilized for DEXA scanning and at the same three sample points for assessment of glucose and insulin dynamics, as previously utilized [18]. FSIGTs were applied before DEXA scanning whenever possible or at least two days after refeeding following the scans to prevent the 24 h food and water withdrawal from impacting FSIGT measurements. A venous catheter (Abbocath, 16ga, Abbott Laboratories, North Chicago, IL) was placed aseptically into a jugular vein approximately one hour prior to collection of the first blood sample on the morning of the FSIGT. A 124.5 cm extension tubing set (Seneca Medical, Tiffin, OH) was attached to the catheter and then secured to the wool of the ewes' backs to allow for infusion and sampling without disturbing the animal. Ewes were maintained in individual adjacent pens with free access to water, but no feed was provided during the test. Baseline blood samples were taken at -15 min and immediately prior to intravenous glucose injection (250 mg/kg BW, 50% dextrose, Vedco Inc., St. Joseph, MO). Blood samples were then taken at 2, 4, 6, 8, 10, 12, 14, 16, and 19 min following glucose injection. At 20 min post-glucose, insulin (20 mIU/kg BW recombinant human insulin, Humulin R, Lilly, Lake Forest, IL) was administered via injection through the catheter and blood sampling continued at 22, 23, 24, 25, 27, 30, 35, 40, 50, 60, 70, 80, 100, 120, 150, 180, 210 and 240 min post-glucose injection as previously described [19,20].

Parameters of the minimal model of glucose and insulin dynamics; insulin sensitivity (SI), glucose effectiveness (Sg), acute insulin response to glucose (AIRg), and disposition index (DI); were determined by simultaneous fitting of glucose and insulin curves resulting from the FSIGT according to the following equations using MinMod Millenium software (Version 5.10, MinMod Inc.) [19,21]:

G'(t) = -(Sg+X)*G(t) + Sg*Gb,

where G(t) = glucose at minute (t) and Gb = baseline glucose

X'(t) = -P_2_*X(t) + P_3_*(I(t)-Ib),

where X(t) = insulin action at minute (t), I(t) = insulin concentration at minute (t), Ib = baseline insulin concentration, P_2 _= loss rate of insulin action (X), P_3 _= action of one unit insulin on glucose disposal per minute

SI represents the acceleration of glucose clearance by the insulin present (SI = P_3_/P_2_), Sg is the basal (unstimulated) glucose clearance rate, AIRg is the initial insulin response available to act on glucose clearance (via SI) measured in the first 10 min following glucose injection, but prior to exogenous insulin administration, and DI is a measure of the absolute insulin action potential attributable to the initial insulin response (AIRg) and the tissue response (SI).

### Fetal and maternal tissue collection

At day 78 × 1 d of gestation, ewes were sedated with Ketamine (22.2 mg/kg body weight) and maintained under isofluorane inhalation anesthesia (4% induction, 1-2% maintenance). Ewes were then exanguinated while under general anesthesia and the gravid uterus quickly removed. There were 5 singleton and 4 twin fetuses from C ewes, 3 singleton and 8 twin fetuses from OW125 ewes and 2 singleton and 6 twin fetuses from OB150 ewes. Fetal BW, CRL, thoracic and abdominal circumferences were recorded for all fetuses. Fetal tissues, including the heart, kidneys, adrenals, pancreas, liver and perirenal fat depots, were dissected out and tissue weights recorded. Fetal hearts were dissected further to record weights of right and left ventricles. A mean weight was calculated for paired organs (kidneys, adrenals and perirenal fat depots). Maternal liver was also collected and weighed.

### Ether extraction of fetal liver

Tissue dry matter (DM) and percent lipid (ether extract) was determined on duplicate 0.5 g samples of tissue by AOAC procedures [22]. Briefly, samples were weighed out onto dried filter paper and the filter paper folded to securely enclose samples. The samples were dried at 100° C for 24 h, then placed into an ether refluxer for 24 h. Weights were recorded between steps and the difference in weights were used to calculate lipid as a percent of DM.

### Biochemical assays

Plasma glucose was measured in triplicate by photoabsorbance following the addition of glucose hexokinase reagent (Liquid Glucose Hexokinase Reagent Set, Pointe Scientific, Inc., Canton MI) using 96-well plates as previously described [23]. Mean intraassay coefficient of variation (CV) was 1.5% and interassay CV was 4.0%. Plasma insulin was measured in duplicate by commercial radioimmunoassay kit (Siemens Healthcare Diagnostics, Deerfield, IL). Intra- and inter-assay CV for insulin were 7.6% and 14.9%, respectively. Serum leptin was measured by commercial radioimmunassay kit (Multi-species Leptin RIA, Millipore Corporation, Billerica, MA) in duplicate within a single assay. Intra-assay CV was 2.5%. Leptin and insulin assays were previously validated for use in sheep [23].

### Statistical analyses

Differences among sample time point or weekly and biweekly measurements (SI, AIRg, % fat, BMI, BW, BCS, basal glucose, insulin and leptin) were assessed using a mixed analysis of variance with repeated measures using SAS (SAS Institute Inc., Cary, NC). There was no significant effect of pen/group on changes in BW or % fat in any treatment group when pen was included in the statistical model; therefore, pen/group was eliminated from analyses and each ewe was considered a single experimental unit. There was no significant effect of dietary treatment on pregnancy type (single vs. twin) (P = 0.35). Also, there was no significant effect of pregnancy type (single vs. twin) on fetal size measures or BW-adjusted organ weights. Therefore, all analyses and data presented are for single and twin fetuses combined. Comparisons of mid-gestation measures (e.g. fetal size measures, fetal organs weights, etc.) were made using analysis of variance by general linearized models in SAS. Regression analysis was used to determine relationships between various maternal and fetal variables. Differences are determined significant at P ≤ 0.05 and trends at P ≤ 0.10.

## Results

### Ewe anthropometrics and organ weights

**Figure 1:**
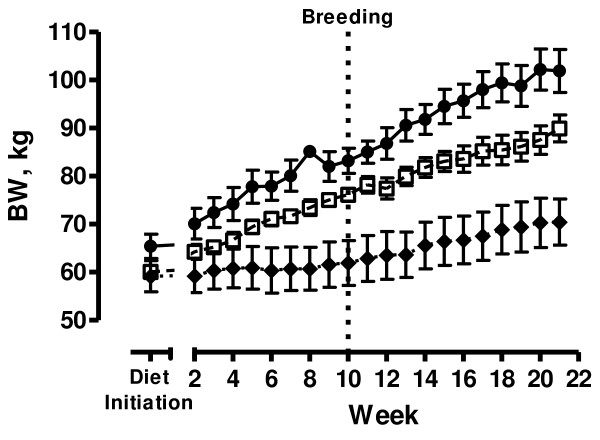
**Body weight**. Body weight (BW) of control (-◆- closed diamonds, fed to requirement; n = 7), overweight (-□- open squares, fed 125% of nutrient requirements; n = 8) and obese (-•- closed circles, fed 150% of nutrient requirements; n = 5) ewes throughout study.

**Figure 2:**
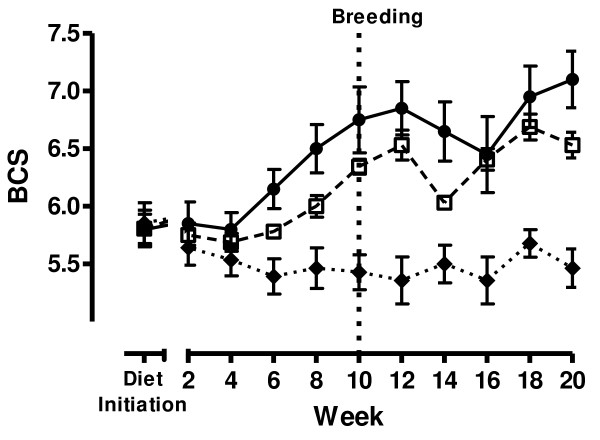
**Body condition**. Body condition score (BCS) control (-◆- closed diamonds, fed to requirement; n = 7), overweight (-□- open squares, fed 125% of nutrient requirements; n = 8) and obese (-•- closed circles, fed 150% of nutrient requirements; n = 5) ewes throughout study.

Ewe BW was not different between any groups at diet initiation (P > 0.10). Weekly BW of ewes increased from diet initiation to mid-gestation in all groups (Figure 1). The C ewes did not increase significantly in BW from diet initiation to breeding (P = 0.07), but exhibited a 14% increase (P < 0.001) in BW during early pregnancy from breeding to mid-gestation. Both OW125 and OB150 ewes increased in BW from diet initiation to breeding (P < 0.01) and from breeding to mid-gestation (P < 0.01). From diet initiation to breeding, OW125 ewes increased in BW by approximately 27.1%, while OB150 ewes increased by 27.8% over the same interval. By mid-gestation, OW125 ewes had increased BW by 49.9% and OB150 ewes increased by 55.8% from BW at diet initiation. Biweekly BCS decreased in C ewes from diet initiation to breeding due to a statistically significant (P < 0.05), yet slight decrease in BCS of approximately 0.5 score unit which occurred in the first several weeks of the study, and maintained a relatively constant BCS thereafter. The OW125 and OB150 ewes increased in BCS from diet initiation to breeding (P = 0.009 and P < 0.001, respectively), but did not increase further during early pregnancy from breeding to mid-gestation (P = 0.34 and P = 0.17, respectively; Figure 2).

Body fat % determined by DEXA revealed a significant treatment by sample point interaction (P < 0.01; Table 2). Body fat % was similar for C, OW125 and OB150 groups before diet initiation, but increased in all treatment groups from diet initiation to mid-gestation. Percentage body fat of C ewes was lower than OW125 ewes (P = 0.02) and OB150 ewes (P < 0.001) prior to breeding and remained lower than both groups at mid-gestation (P < 0.001 for each). The OB150 ewes had a higher % body fat than OW125 ewes prior to breeding (P = 0.03), but not in mid-gestation due to a marked increase in % body fat of OW125 ewes. Ewe % body fat was strongly associated with fetal BW-adjusted perirenal fat mass (R^2 ^= 0.64, P < 0.001) at mid-gestation. Ewe BMI was strongly associated with % body fat (R^2 ^= 0.79, P < 0.01), more robustly than the relationship of BCS to % body fat (R^2 ^= 0.37, P < 0.01). Thus, ewe BMI of C, OW125 and OB150 ewes showed changes similar to those of % body fat from diet initiation to mid-gestation (Table 2). However, BMI failed to detect differences between OW125 and OB150 ewes prior to conception and also between breeding and mid-gestation in the OW125 ewes (Table 2). At necropsy in mid-gestation, ewe liver weight was significantly affected by treatment (P < 0.01) due to increased total liver weight with increased level of feeding (C: 766 × 5.7 g < OW125: 1061 × 6.0 g < OB125: 1162 × 6.6 g), but this effect was eliminated when liver weight was adjusted for ewe BW.

**Table 2:** Ewe body mass index (BMI) and % body fat of subset of ewes utilized for DEXA

	Control, n = 4		OW125, n = 4		OB150, n = 4	
*Sample point *	*Mean *	*SE *	*Mean *	*SE *	*Mean *	*SE *
***BMI, cm/kg^2 ^***						
**Diet initiation**	31.9^a,A^	1.4	34.0^a,A^	0.8	34.0^a,A^	1.7
**Breeding**	35.9^a,AB^	2.3	43.7^b,B^	1.3	44.3^b,B^	3.6
**Mid-gestation**	38.6^a,B^	2.7	47.4^b,B^	2.2	53.2^b,C^	1.7
***Body fat, %***						
**Diet initiation**	4.6^a,A^	0.5	4.5^a,A^	0.2	4.5^a,A^	0.3
**Breeding**	11.1^a,B^	2.4	16.2^b,B^	1.0	21.1^c,B^	2.4
**Mid-gestation**	16.2^a,C^	1.5	25.1^b,C^	1.7	27.0^b,C^	1.2

Superscripts in lower case indicate significant differences (P < 0.05) between treatment groups within a sample point (across rows) and upper case indicate differences (P < 0.05) between sample points within a treatment group (down columns). Number of observations (n) denoted represents number of ewes.

### Biweekly blood variables

**Figure 3:**
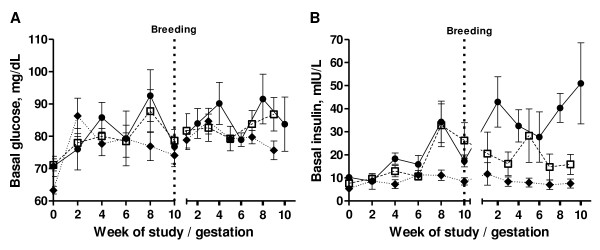
**Biweekly plasma glucose and insulin concentrations**. Biweekly plasma glucose (A) and insulin (B) concentrations control (-◆- closed diamonds, fed to requirement; n = 7), overweight (-□- open squares, fed 125% of nutrient requirements; n = 8) and obese (-•- closed circles, fed 150% of nutrient requirements; n = 5) ewes through the pre-pregnancy portion of the study prior to breeding (dashed line) and through the first half of gestation.

Baseline blood glucose concentrations increased over time between diet initiation and breeding (P < 0.01; Figure 3A); however, there was no significant effect of treatment on biweekly glucose concentrations during this period (P = 0.29). There were no further changes in glucose concentrations in any groups between breeding and mid-gestation. Baseline insulin concentrations increased in OW125 and OB150 ewes between diet initiation and breeding (treatment*week interaction, P = 0.05), and remained elevated between breeding and mid-gestation (Figure 3B). In contrast, baseline insulin concentrations of C ewes remained relatively low and constant from diet initiation to mid-gestation. Insulin concentrations at mid-gestation were significantly associated with fetal BW-adjusted perirenal fat depot weight (R^2 ^= 0.22, P = 0.02) and fetal BW-adjusted pancreas weight (R^2 ^= 0.19, P = 0.03). Leptin concentrations increased in OW125 and OB150 ewes from diet initiation to breeding, but did not change in C ewes. From breeding to mid-gestation, leptin concentrations did not increase further in OW125 ewes, but continued to increase in OB150 ewes (Table 3). Maternal leptin at mid-gestation was associated with BW-adjusted weights of fetal liver and pancreas (R^2 ^= 0.39 and R^2 ^= 0.28, respectively; P < 0.01 for each).

**Table 3:** Ewe leptin concentrations throughout the experimental period

	Control, n = 7		OW125, n = 7		OB150, n = 4	
*Sample point *	*Mean *	*SE *	*Mean *	*SE *	*Mean *	*SE *
***Leptin, ng/mL ***						
**Diet initiation**	1.66^a,A^	0.14	1.95^a,A^	0.10	1.90^a,A^	0.26
**Breeding**	2.29^a,A^	0.21	6.76^b,B^	0.78	7.47^b,B^	1.21
**Mid-gestation**	3.04^a,A^	0.42	6.22^b,B^	1.23	11.29^c,C^	2.38

Superscripts in lower case indicate significant differences (P < 0.05) between treatment groups within a sample point (across rows) and upper case indicate differences (P < 0.05) between sample points within a treatment group (down columns). Number of observations (n) denoted represents number of ewes.

### Intravenous glucose tolerance tests

**Figure 4:**
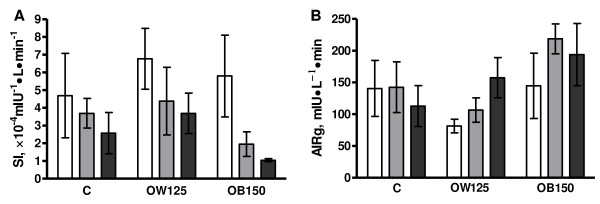
**Insulin sensitivity and acute insulin response**.Insulin sensitivity (SI; A) and acute insulin response to glucose (AIRg; B) at the three different sample points: 1) immediately prior to diet initiation (white bars), 2) immediately prior to breeding (gray bars) and 3) at mid-gestation (black bars) for a subset of control (C, n = 4), overweight (OW125, n = 4) and obese (OB150, n = 4) ewes. There was an overall effect of time point on SI (P = 0.04) and an overall trend for treatment effect on AIRg (P = 0.06).

While there was no overall effect of treatment on SI, SI did decrease overall from diet initiation to mid-gestation (P = 0.04; Figure 4A). With all treatment groups combined, SI at diet initiation tended to differ from SI prior to conception (P = 0.07) and differed significantly from mid-gestation (P = 0.02). SI did not change significantly from breeding to mid-gestation (P = 0.49). Within OB150 ewes only, SI tended to decline across time points (P < 0.09). In OB150 ewes only, there was a significant relationship between SI and % body fat (R^2 ^= 0.40, P = 0.03). AIRg (β-cell responsiveness) was affected by treatment (P = 0.06) due to greater AIRg in OB150 ewes than C ewes (P = 0.09) and OW125 ewes (P = 0.02; Figure 4B). Disposition index, the product of SI and AIRg, was not significantly affected by sample time point or treatment.

### Fetal morphometrics and organ characteristics

Mean fetal weight at midgestation tended (P = 0.09) to increase with increasing level of feeding (C < OW125 < OB150) (Table 4). Similarly, fetal crown rump length, thoracic girth and abdominal girth were all increased in the overfed ewes. Crown rump length was greater in OW125 (P = 0.02) and OB150 (P = 0.02) than in C fetuses, but no significant difference was observed between OW125 and OB 150 fetuses (P = 0.76). Fetal thoracic and abdominal girths were greater in OB150 fetuses than C or OW125 with no significant differences between the latter groups (Table 4). Absolute fetal organ weight was greater (P ≤ 0.05) in OB150 fetuses than C or OW125 fetuses for heart, kidneys, adrenals and pancreas (Table 5). Fetal organ weights were increased (P ≤ 0.05) in both OW125 and OB150 groups, relative to C, for right and left ventricles and perirenal fat. Fetal liver weight was greater (P = 0.03) in OW125 than C fetuses, and greater still (P = 0.03) in OB150 versus OW125 fetuses. For kidneys and adrenals, these treatment effects were eliminated when organ weights were adjusted for fetal BW. For BW-adjusted organ weights of the heart, pancreas and liver, OB150 weights were greater (P ≤ 0.05) than C or OW125, the latter not being different from each other. Fetal BW-adjusted right ventricle weight was greater in OB150 fetuses than C fetuses, but OW125 weights, which were intermediate, were not significantly different from C or OB150 ewes. BW-adjusted tissue weights of the left ventricle and perirenal fat depots were not different between OW125 and OB150 fetuses which were both greater than C fetuses (P ≤ 0.05; Table 5).

**Table 4:** Fetal body weight and morphometrics on day 78 of gestation

	Control, n = 9		OW125, n = 11		OB150, n = 8	
	*Mean *	*SE *	*Mean *	*SE *	*Mean *	*SE *
**Fetal weight, g**	291.5	9.97	311.8	9.20	324.4	9.60
**Crown-rump length, cm**	21.4^a^	0.51	22.9^b^	0.51	23.6^b^	0.26
**Thoracic girth, cm**	13.8^a^	0.16	14.1^a^	0.16	14.9^b^	0.07
**Abdominal girth, cm**	12.8^a^	0.42	13.0^a^	0.22	14.4^b^	0.35

Differing superscripts across rows indicate significant differences, P < 0.05. Number of observations (n) denoted represents number of fetuses.

**Table 5:** Fetal organ weights on day 78 of gestation

	Organ weight, g						(Organ weight/fetal weight)*100					
	Control, n = 9		OW125, n = 11		OB150, n = 8		Control		OW125		OB150	
	*Mean *	*SE *	*Mean *	*SE *	*Mean *	*SE *	*Mean *	*SE *	*Mean *	*SE *	*Mean *	*SE *
**Heart**	2.42^a^	0.10	2.64^a^	0.13	3.26^b^	0.19	0.86^a^	0.02	0.84^a^	0.04	1.01^b^	0.08
**Right ventricle**	0.59^a^	0.05	0.77^b^	0.04	0.92^b^	0.04	0.21^a^	0.02	0.26^ab^	0.02	0.29^b^	0.02
**Left ventricle**	0.73^a^	0.07	1.04^b^	0.05	1.16^b^	0.13	0.26^a^	0.03	0.33^b^	0.02	0.36^b^	0.03
**Kidney**	1.56^a^	0.06	1.55^a^	0.06	1.83^b^	0.16	0.54^a^	0.01	0.50^a^	0.02	0.56^a^	0.04
**Adrenal**	0.05^ab^	0.00	0.05^a^	0.00	0.06^b^	0.00	0.02^a^	0.00	0.02^a^	0.00	0.02^a^	0.00
**Pancreas**	0.30^a^	0.03	0.38^a^	0.03	0.60^b^	0.09	0.10^a^	0.01	0.12^a^	0.01	0.18^b^	0.03
**Liver**	16.67^a^	0.42	18.68^b^	0.71	20.88^c^	0.76	5.75^a^	0.16	5.98^a^	0.09	6.45^b^	0.21
**Perirenal fat**	0.51^a^	0.04	0.79^b^	0.04	0.82^b^	0.06	0.17^a^	0.01	0.26^b^	0.01	0.25^b^	0.01

Differing superscripts indicate significant differences (P < 0.05) across rows, but within category (organ weight or organ weight/fetal weight). Number of observations (n) denoted represents number of fetuses.

Lipid content of fetal liver was also affected by maternal dietary treatment (P < 0.001). OB150 fetal livers had greater % lipid (9.6 × 0.89%) than in livers of C (4.1 × 0.85%) or OW125 (6.4 × 0.76%) fetuses (P < 0.03). Hepatic % lipid was not significantly different between C and OW125 fetuses (P = 0.13).

## Discussion

To our knowledge, this study is the first to assess changes in maternal glucose and insulin dynamics, BCS and absolute % body fat, along with fetal growth and organ development, under two different levels of overfeeding beginning prior to and continuing throughout the first half of gestation in a large precocial species. Sheep are common models for studying fetal development because the timeline of fetal organ development and physiologic responses is similar between sheep and human [24]; however, other ewe models of maternal overnourishment or high glycemic intake have not applied feeding treatments until after conception [25,26] or very late in gestation [27-29] which is less relevant to the problem of already overweight or obese women becoming pregnant. Our model is unique in that it establishes obesity induced by overfeeding beginning 10 weeks prior to conception, thus allowing examination of the effects of the maternal overweight and obese condition beginning prior to conception and continuing throughout gestation. In this study, the differing levels of overfeeding resulted in progressively increasing maternal body weights and % body fat, which corresponded to progressively increasing fetal size and differential increases in fetal heart, liver, pancreas and perirenal fat mass, as well as fetal hepatic lipid content at mid-gestation, which corresponds to mid-gestation in humans.

The OW125 ewes, which entered pregnancy with % body fat approximately 5% lower than OB150 ewes, failed to induce significant differences in fetal thoracic girth, abdominal girth or fetal BW-adjusted heart, liver and pancreas weights relative to C ewes and their fetuses at midgestation even though % BW gain in OW125 ewes was similar to OB150 ewes prior to pregnancy. Therefore, as indicated by other investigators, pre-gravid body fat, and not body weight, may be the best indicator of risk for altered fetal development [30]. Since BW-adjusted fetal weights of heart, pancreas and liver were only increased in OB150 ewes, these organs may be protected from overgrowth with moderate maternal overfeeding and pre-pregnancy adiposity, as seen in OW125 ewes. Similar perirenal fat mass of OW125 fetuses to OB150 fetuses suggests excess energy substrate preferentially stores as visceral fat and that heart, pancreas and liver development is only affected when fetal substrate delivery surpasses the ability of fetal fat depots to incorporate additional substrate. The greater degree of fetal overgrowth and increased BW-adjusted fetal organ weights (heart, pancreas and liver) observed in OB150 fetuses suggests that a greater amount of excess energy substrate was redistributed to feto-placental tissues of OB150 animals, fueling fetal growth instead of continued maternal fat deposition in an animal already having high fat stores. This may overburden fetal metabolism with glucose in excess of developmental need and beyond what can be stored as fat, causing the altered organ development observed in the OB150 fetuses. Supporting this hypothesis, maternal % body fat was a good predictor of fetal perirenal fat mass in this study, indicating the increasing incorporation of excess substrate into fetal intra-abdominal fat with increasing maternal fat mass.

A redistribution of maternal excess energy substrate to the fetus is likely driven by insulin resistance of maternal tissues [31]. While there was no significant effect of treatment on SI in this small subset of animals, the mean SI of OB150 ewes (1.04 × 0.09 × 10^-4^mIU^-1^·L·min^-1^) was similar to values reported for type II diabetic men and women (0.74 × 0.3), whereas SI in C (2.57 × 1.2) and OW125 (3.69 × 1.1) ewes was more comparable to SI observed in lean (4.89 × 0.7) and non-diabetic obese (2.75 × 0.5) subjects [32]. Furthermore, SI at mid-gestation for OB150 ewes was within the second lowest reference quintile developed for SI in apparently healthy horses (SI range 0.79-1.5), but fell into the upper fourth (2.28-3.04) and fifth (3.05-5.94) equine references quintiles in C and OW125 ewes, respectively [33]. The relationship between SI values measured using minimal model analysis in sheep versus horses or humans has not been determined, but such comparisons provide a point of reference for discussing values determined in different species. The degree of pre-pregnancy adiposity may determine how early in gestation maternal insulin resistance develops to a level which sufficiently slows maternal energy storage/utilization and enhances fetal nutrient delivery. This agrees with observations that pre-pregnancy BMI in women is an important indicator for gestational diabetes mellitus (GDM), pre-eclampsia and fetal macrosomia [30,31].

Greater lipid content of OB150 fetal livers likely accounts for part of the increased fetal liver weight observed in this organ. Non-alcoholic fatty liver disease is characterized by adipose accumulation and inflammatory stress in the liver and is associated with development of the metabolic syndrome. Though few studies have evaluated the effects of maternal nutrition on fetal and postnatal liver function, high fat feeding has been shown to result in increased postnatal hepatic fat content in rats and altered gluconeogenic enzymes and hepatic fat content in fetal livers of nonhuman primates [34,35]. Increased visceral adiposity has also been shown to be a strong predictor of fatty liver [36]. Thus, the combination of increased fetal hepatic lipid content and greater visceral (perirenal) fat in OB150 fetuses may play a role in predisposing these fetuses to postnatal development of metabolic disease. In response to intrauterine growth restriction induced by placental insufficiency in ewes, fetal liver growth was reduced and gene expression of pathways affecting nutrient sensing, insulin responsiveness and gluconeogenesis were altered [37]. Hepatic overgrowth and/or fatty liver may affect similar pathways, but further research into the functional changes occurring during hepatic overgrowth induced by maternal overnourishment and obesity is needed.

An enlarged pancreas in fetuses of obese ewes have also been shown to have increased insulin content and number of insulin-producing cells in studies using the same experimental paradigm as the present study comparing only obese (analogous to OB150) and control treatments [17]. These alterations in pancreas size and composition provide a mechanism for the fetal programming of β-cell function and future metabolic disease by maternal overnourishment/obesity [31].

Maternal leptin was significantly associated with BW-adjusted weights of fetal liver and pancreas, indicating a potential role of leptin in predicting risk for altered development of these important fetal organs. Furthermore, increased maternal insulin was associated with increased fetal pancreas and perirenal fat mass. Since the pancreas and liver are both organs involved in glucose metabolism, altered fetal development of these organs is likely particularly important in conferring future risk for obesity and metabolic disease to these offspring. Fetal adrenals and kidneys, organs important for stress responses and blood pressure regulation, appeared to grow proportionally to the fetal body, suggesting these organs may be less affected by maternal adiposity and dietary excess prior to pregnancy and during early gestation.

During early pregnancy, both overfed groups gained, on average, an additional 0.2 (OW125) or 0.3 (OB150) BCS units, which suggests that additional fat accumulation was similar. However, % body fat gain in OW125 ewes by DEXA from pre-conception to mid-gestation was enough to result in similar % body fat at the end of the study in both overfed groups. Thus, during early pregnancy, OW125 may have gained more intra-abdominal fat (indicated by increased overall fat with DEXA), without substantial change in subcutaneous fat (assessed by BCS), indicating the importance of comprehensive measures of body composition such as those provided by DEXA. Also during the early pregnancy period, C ewes increased in BW, maintained a moderate BCS and increased in % body fat, implicating intra-abdominal fat accumulation for increased adiposity without a change in subcutaneous fat. This observation is consistent with the tendency for visceral fat accumulation during pregnancy in women [38]. Increased intra-abdominal fat provides a useful energy store in preparation for the increased energy demands of late gestation and lactation. However, intra-abdominal fat is also associated with the development of insulin resistance and other disease risk in non-pregnant subjects due to its greater metabolic and endocrine activity [39-41]. Therefore, excessive intra-abdominal adiposity in gestation may increase risk for gestational diabetes [42]. Surgical removal of visceral fat 4 wks prior to breeding was associated with improved overall insulin sensitivity and improved suppression of hepatic glucose production (hepatic insulin sensitivity) in late gestation in the rat, further supporting the importance of visceral adiposity in determining the degree of insulin resistance developed in pregnancy [43]. While DEXA analysis may be a less practical assessment of maternal fat accumulation relative to BW and BCS, methods that account for central adiposity have been shown to be better predictors of perinatal outcomes in women as they do in the present study in the pregnant ewe [44].

## Conclusions

We have presented evidence of hyperinsulinemia, hyperleptinemia, greater fetal growth and altered fetal organ development in OB150 ewes overfed to achieve 21% body fat prior to conception compared to OW125 ewes fed to 16% body fat or C ewes fed to 11% body fat prior to conception. Thus, fetal development of heart, liver and pancreas may be particularly sensitive to pre-conception and/or early gestational changes in maternal body composition and metabolism beyond what is compensated for by fetal visceral fat deposition, developmental changes which may predispose to postnatal metabolic disease However, greater perirenal adipose mass in OW125 and OB150 ewes suggests that OW125 fetuses may still be at risk for future development of obesity and metabolic complications. Further study of fetal organogenesis throughout the remainder of gestation in this model is justified to fully clarify the effects of varying levels of maternal nutritional excess and obesity on late fetal development and the postnatal consequences of altered development. Our findings support those observed in pregnant women; however, our ewe model offers the advantage of utilizing DEXA for objective measures of fat mass, a technique not used in pregnant women due to the perceived potential risk to fetal well-being, as well as assessment of mid-gestational fetal size and organ development [44]. Overall, level of maternal overfeeding and adiposity prior to pregnancy has a significant impact on degree of fetal overgrowth and on alterations observed in fetal organ development at mid-gestation, with more moderate maternal adiposity resulting in much less severe changes in fetal development at mid-gestation than in more obese dams.

## Competing interests

The authors declare that they have no competing interests.

## Authors' contributions

LAG, PWN and SPF designed the study. LAG and ABU performed the research/data collection with assistance from NML, LZ and SPF. LAG, NML, LZ and YM conducted laboratory analyses. LAG performed data analyses, interpreted data and drafted manuscript. PWN and SPF provided financial support and significant editing of the manuscript. All authors read and approved the final manuscript.
